# Small and Medium Enterprises’ Perspectives on Food Fortification Amid the Growing Burden of Malnutrition

**DOI:** 10.3390/nu14183837

**Published:** 2022-09-16

**Authors:** Jimena Monroy-Gomez, Chiara Ferraboschi, Kesso Gabrielle van Zutphen, Breda Gavin-Smith, Daniel Amanquah, Klaus Kraemer

**Affiliations:** 1 *Sight and Life*, P.O. Box 2116, 4002 Basel, Switzerland; 2 Department of Human Nutrition & Health, Wageningen University & Research, 6708 PB Wageningen, The Netherlands; 3 Department of International Health, Johns Hopkins School of Public, Baltimore, MD 21218, USA

**Keywords:** food fortification, food industry, small and medium enterprises, food systems, public health, micronutrient deficiencies, burden of malnutrition, nutrient profiling, public–private partnership

## Abstract

The need for a profound food system transformation has never been greater. The growing burden of malnutrition has become the new normal, with two billion people who are overweight, over 140 million children under five who are stunted and over two billion people affected by hidden hunger. Food fortification has been recognized as a cost-effective strategy to address micronutrient deficiencies. Small and medium enterprises (SMEs) play a strategic role in the food supply chain in low- and middle-income countries, accounting for over 80% of food sales. It is therefore critical to create an enabling environment to facilitate SMEs’ involvement in food fortification practices as a potential solution to tackle all forms of malnutrition. This review highlights SMEs’ relevance as agents of change in the food system through food fortification practices and their indirect yet key role in producing nutritious, tasty and affordable foods. It discusses their challenges (e.g., access to long-term finance, sustainable technical assistance, limited capacity), presents solutions and discusses how different actors can help SMEs to overcome these challenges. Furthermore, it presents a relevant public–private partnership case study to demonstrate how SMEs can address the growing burden of malnutrition through food fortification practices, nutrient profiling schemes and demand generation.

## 1. Introduction

The burden of malnutrition represents a global threat, as two billion people are obese and 140 million children under five are stunted [1]. In addition, 43% of children are anemic [2] and 30% are vitamin A deficient [3]. Food fortification is considered the most cost-effective strategy to prevent micronutrient deficiencies [4,5] and was included in the 10 recommended double-duty actions (DDA) established by the World Health Organization to address growing malnutrition rates [6,7]. The central pillar of DDA consists of the “no harm” approach, which implies that tackling one type of malnutrition (e.g., micronutrient deficiencies and underweight) should not increase another form of malnutrition (overweight and obesity) [8]. In low- and middle-income countries (LMICs), rapid urbanization and the nutrition transition are increasing the demand for, and consumption of, ultra-processed food, which tends to be energy-dense and nutrient-poor food [9] and is one of the risk factors for overweight and obesity [10]. Therefore, clear nutritional criteria for producing food in LMICs must be established when designing and implementing food fortification strategies [11] to provide nutrient-rich foods and help tackle all forms of malnutrition. Small and medium enterprises (SMEs) should be supported to follow the DDA approach because of their key role in producing, processing and distributing food along the value chain in LMICs. Indeed, in LMICs, SMEs provide around 80% of food commodities [12,13] and about 40–70% of employment [13,14,15]. However, they have received little attention and still face challenges that hinder them from being considered as key agents of change to improve the nutritional situation in LMICs. This review aims to highlight the most pressing challenges faced by SMEs in the production and distribution of fortified foods. It also describes possible solutions and actors that could enable the environment for SMEs to overcome these challenges.

First, this review discusses the crucial role that SMEs play in the food value chain and economic development in LMICs. It showcases examples of SMEs involved in the production and distribution of food that have successfully incorporated a social business approach to improve nutrition outcomes. Second, this review describes the challenges that SMEs face when launching fortified products in the market, and puts forward several solutions, including a public–private partnership (PPP) case study, that could help overcome these challenges.

The methodology of this review consisted of two research stages: a literature review and interviews with experts working in SMEs and social businesses in LMIC. In the first stage, the authors searched databases for empirical articles to identify previous publications related to the topic. Since the literature is limited, a cutoff date was not set, and authors considered articles in English published in scientific databases, grey literature and reports from organizations working in the field. The databases used were PubMed, Web of Science, Google Scholar and Research Gate. The research terms used were “small and medium enterprises”, OR “SME” OR “SMEs” AND “Social Business” or Social Businesses” AND “Low- and middle-income countries” OR “LMIC” OR “LMICs” AND “Nutrition” OR “Nutritional interventions” AND “Challenges” OR “Barriers”. In the second stage of the review, the authors conducted interviews with experts from *Sight and Life* (Obaasima project), DSM (micronutrient premixes knowledge), GRET (MERIEM project) and HarvestPlus to better understand the challenges SMEs and social businesses face while implementing food fortification initiatives. The interviews also served as a source of information when available evidence was limited.

## 2. The Role of SMEs as Agents of Change in LMICs

SMEs (defined as independent businesses with a maximum of 250 employees) are potential agents of change for sustainable transformation in the food system [13]. Especially in LMICs, these enterprises can benefit the most vulnerable by reducing economic inequalities, creating new jobs and facilitating economic development. In addition, SMEs can help to improve social issues, for example, by offering services and nutritious products that benefit the public [16]. Indeed, formal SMEs are essential avenues for local employment, accounting for 40–70% of the agri-food sector employment in LMICs [13,14,15], and distributing up to 80% of commodities in the food value chain [12,13]. Informal SMEs are equally important sources of food and livelihood as they are the main food distribution channel [15,16]. In Asia, formal and informal small and medium food processors account for 60% of gross domestic product, providing more than 90% of employment [17]. In Africa, SMEs provide more than 80% of commodities in the continent and 65% of employment in rural areas [13]. SMEs are also heavily involved in the production and distribution of food. The number of SMEs involved in these operations in Africa has rapidly increased and will continue to grow [15,18]. This strong and growing presence underlines their potential to influence social concerns, such as economic development, local food production and the consumption of nutritious food amid the burden of malnutrition in LMICs. More than ever, SMEs represent a crucial actor in the food system. The COVID-19 pandemic and the war in Ukraine have affected country-to-country food trades, and there have been food shortages and food insecurity across different regions [19]. Additionally, food prices are expected to rise because of the war in Ukraine, and people will be unable to obtain sufficient staple foods and nutritious diets [19]. Consequently, the burden of malnutrition is expected to increase [20,21,22,23].

A potential solution that can address social issues (e.g., malnutrition) and offer business advantages is the social business model (SBM) approach. Social businesses aim to solve social issues, provide sustainable functionality and empower entrepreneurs—and also improve the return on their investments [24,25]. Social businesses can play a crucial role by taking different approaches to supply, and create demand for, nutritious products [18]. Below, we present two examples that showcase how SMEs have implemented an SBM approach by producing and distributing nutritious foods to tackle different forms of malnutrition in LMICs.

La Laiterie du Berger (LDB)—a Senegalese small social business that produces dairy products—has launched an innovative SBM with local farmers. This SBM aims to reduce the variability in milk supply, maintain regular production of dairy products and improve the nutritional status of farmers’ children [26]. In the model implemented by LDB, the children of farmers who supply a certain amount of milk to LDB receive free, micronutrient-fortified yogurt during the dry season. Additionally, a social behavioral change communication campaign was conducted with the farmers’ households to expand the demand for, and distribution of, nutritious and fortified foods [27]. This innovation ensures a steady milk supply and the continued production and availability of fortified dairy products during the dry season.

Another example is the MERIEM (mobilizing Sahelian businesses for innovative, large-scale responses to fight malnutrition) project, which aims to offer commercial solutions to tackle malnutrition among children in Burkina Faso, Mali and Niger. The project encourages local businesses to develop innovations and distribute fortified food solutions at an affordable price. Funded by the Agence Française de Développement (AFD) and the Bill & Melinda Gates Foundation, the MERIEM project supports local social businesses by providing technical advice through a panel of experts from different organizations including GRET, Hystra, the Institut de Recherche et d’Applications des Méthodes de Développement (IRAM), Initiatives Conseil International (ICI), the Institut de Recherche pour le Développement (IRD), Ogilvy Change and ThinkPlace. Together, they also create social marketing campaigns to increase the awareness of exclusive breastfeeding and proper feeding practices during the first 1000 days [28]. To date, several fortified products have been launched under this SBM, including fortified flour (*Super Léo*), fortified milk (*Foura Soga*) and fortified spices (*Nafama*) by the MERIEM project. Within the framework of this project, GRET used its experience in SBM, proven in particular in Madagascar with the development of the fortified infant flour *Koba Aina*, developed more than ten years ago and distributed since 2013 by the Malagasy social enterprise Nutri’zaza [28,29,30,31].

As the above examples show, these SMEs were able to incorporate SBM approaches to help tackle micronutrient deficiencies through food fortification practices while maintaining their business objective. However, SMEs still face an extensive list of challenges when producing fortified food, the most critical of which are discussed in the following section.

## 3. Challenges Faced by SMEs Implementing Food Fortification

Despite the relevant role SMEs play in the production and supply of fortified foods in LMICs, they face major challenges related to capacity building and still have limited recognition as crucial actors in tackling all forms of malnutrition [15]. This section describes the main challenges SMEs face. First, the lack of data at the upstream level is described and how this overarching challenge limits the design and implementation of food fortification strategies adapted to different country contexts. Second, this review deep dives into challenges that directly affect SMEs. Finally, it describes how the lack of capacity limits the ability of SMEs to scale up food fortification solutions.

### 3.1. Data Gap

Worldwide, there are limited data available about the status of micronutrient deficiencies at individual and population levels, especially in LMICs. The lack of relevant data to design context-appropriate food fortification strategies is an indirect obstacle to SMEs engaging in food fortification practices. This data gap limits the opportunity to guide SMEs to implement food fortification activities according to the local needs. Furthermore, it limits their potential to contribute to the reduction in malnutrition. In addition, data collection on nutritional status is crucial at the upstream level for decision makers to develop public health policies, voluntary standards and mandatory regulations for food fortification based on local needs [32,33,34]. Finally, because of the lack of monitoring, impact evaluation and reporting among food fortification programs within large- and small-scale production facilities, it is difficult to perform accurate implementation assessments. For instance, to evaluate the extent to which food fortification actions have been implemented cost-effectively or to identify actions that have not efficiently addressed nutritional gaps in LMICs [34].

### 3.2. Funding

Limited access to sustainable finance is a crucial barrier that prevents SMEs from implementing innovations or improving their capacity to produce more fortified foods [35]. The cost of manufacturing fortified food compared with that of non-fortified food can be <1% up to 1.33% higher for wheat flour, milk and edible oil [36], and up to 4.5% higher for some varieties of rice [37]. The increase in cost depends on the food commodity, the amount, type and availability of ingredients, the price of micronutrient premixes, and the variable and fixed costs of the SMEs’ operation [36]. Consequently, SMEs typically rely on non-profit organizations for financial support or their own family resources to implement food fortification [38] because bank loans are more likely to be offered to large enterprises across all regions and production sectors. Interestingly, the regions that have more financial gaps are Latin America and the Caribbean, the Middle East and North Africa [38], regions that are affected by micronutrient deficiencies. On the other hand, there is a co-dependence between funds and technical support. For instance, if a sustainable financial model is not in place, technical support is futile as SMEs will be constrained by a lack of investment and unable to apply proper food fortification practices [39].

### 3.3. Technical Support

The lack of technical knowledge limits SMEs’ ability to improve their operation, revenues and shelf storage process [40]. Knowledge gaps in production, nutrition and business are barriers to SMEs optimizing their resources, improving their manufacturing techniques by adding adequate vitamins and minerals (i.e., the correct quantity and type, and at the correct time), and enhancing their business outcomes. Micronutrient fortification must be cautiously incorporated; for example, it is important to consider the type of compounds added to different food vehicles and the bioavailability of the compounds [41]. Depending on the food vehicle and the processing method, SMEs involved in the processing stage must consider at which step of the manufacturing process to add the vitamin and mineral premixes. Moreover, SMEs need technical support to choose the correct type of premix to maintain stability against physical and environmental factors (e.g., temperature, humidity). Additionally, they need to measure the micronutrient content of their final products to verify they meet fortification standards [42] and comply with strict international and local food fortification guidelines. Finally, SMEs are also challenged by a lack of knowledge about the long-term vision for business development, brand positioning expertise and affordable but safe packaging.

### 3.4. Regulation

It is known that national fortification standards often present a challenge to SMEs [39,42]. For instance, in Uganda, the national fortification law only applies to small enterprises with the capacity to process a certain number of tons of maize and oil per day [18]. In Indonesia, fines are imposed when producers do not meet mandatory salt iodization standards [42]. Moreover, SMEs report that the high tax rates for premixes are one of the main barriers to fortification practice. For example, the regulation of taxation for premixes varies globally and highly influences a product’s final price. In Peru, Guatemala and Japan, the tax rate for premixes is zero. In countries such as Mexico, Brazil, Colombia, Vietnam and India, the tax rate varies from 0% to 16%. Africa has the highest tax rate for premixes, yet it is one of the regions most impacted by micronutrient deficiency worldwide; for instance, the tax rate for premixes in Ghana ranges from 38% to 40% [43,44]. Finally, in Bangladesh, the adequate fortification of cooking oil is hampered by the incorrect and misleading labelling of fortified products by producers and inconsistent law enforcement [45].

### 3.5. Capacity Building and Scale-Up

Capacity building refers to the process of developing and strengthening the skills, abilities, processes and resources in an organization [46]. Capacity constraints can vary among SMEs. For instance, some SMEs may not be able to produce high volumes of fortified products. Other SMEs may struggle to expand their market penetration in new locations or types of outlets (e.g., open markets, modern or business-to-business trade). Furthermore, some SMEs may struggle to obtain capital to invest in premixes and machinery, or their business knowledge may be insufficient to increase their profits. In general, increasing SMEs’ capacity increases the possibility of food fortification practices being scaled up in LMICs.

**Figure 1:**
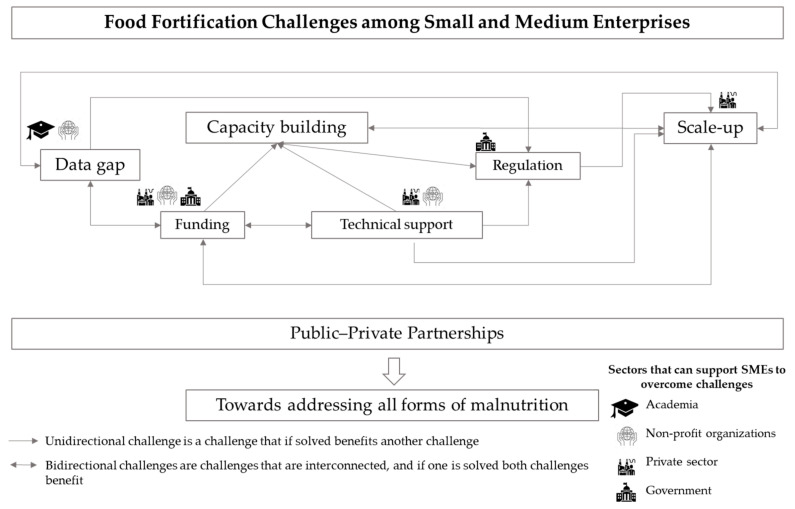
Overview of interconnected food fortification challenges and potential actors to overcome food fortification challenges towards the burden of malnutrition.

However, a challenge can either exist in isolation or co-exist with other challenges (Figure 1). For example, the lack of funds to buy supplies (e.g., premixes, ingredients) and also the machinery required for sophisticated manufacturing techniques is a significant barrier to increasing production and building capacity [41,45]. In addition, a scarcity of funds to buy laboratory assessment tools prevents SMEs from being able to verify nutritional composition and comply with standards [34,42,47]. Thus, scaling up depends on the ability of SMEs to build capacity. Even though SMEs face many barriers to the production of fortified foods, numerous solutions can be implemented by supporting SMEs at different stages across the value chain and by bringing together multidisciplinary expertise. The following section describes different actors and solutions to help SMEs overcome the main challenges of food fortification.

## 4. Actors and Solutions to Help SMEs Overcome Food Fortification Challenges

Collective and multisectoral support is crucial to scale up food fortification practices among SMEs. This section proposes how different actors can help SMEs to overcome the aforementioned challenges.

First, unless data gaps are addressed, it will remain difficult for SMEs to adopt efficient fortification practices that address context-specific micronutrient deficiency. As a trusted source of scientific evidence, academia plays a strategic role in collecting, analyzing and providing data. Once these nutritional data gaps have been filled, the new information could help to inform government policies and standards, and also food companies. Consequently, SMEs will then be able to invest time and resources to develop and implement evidence-based food fortification interventions [34]. In addition, academia must participate as the essential link among the various food chain actors. It could also implement a systematic monitoring tool to track data on nutritional status, cost-effectiveness and efficiency indicators across time, and encourage multiple stakeholders to provide data based on their experiences. Along with academia, non-governmental organizations and the private sector could collect, report and monitor nutritional indicators, and then share their experiences and learnings openly with stakeholders to identify the best way to address the food fortification gaps [35,48]. Examples of this practice include: the Global Fortification Data Exchange, which is a collaboration between the Food Fortification Initiative, Global Alliance for Improved Nutrition, Iodine Global Network and Micronutrient Forum [49]; the Micronutrient Data Innovation Alliance (DInA), launched by the Micronutrient Forum along with other stakeholders [50]; and a free-access online platform that integrates a Global Nutrition and Health Atlas developed by Tufts University in collaboration with Nestlé Research Center [51]. However, to create open data collaborations, interests must be clearly communicated, and the collaborators must be aligned with common goals. Additionally, confidentiality agreements must be implemented between the different actors if required. The Structural Genomics Consortium project is a good example whereby this type of agreement between pharmaceutical companies and academia is feasible and has led to high-quality evidence by validating research and creating knowledge across the field [52].

On the other hand, to address funding challenges, international organizations can provide SMEs with direct funding to buy machinery and also technical support to improve their productivity [53]. An example of direct funding to increase SMEs’ capacity building for food fortification is Sanku—an initiative of the World Food Program Innovation Accelerator. Sanku offers technical training to small-scale flour mills, subsidizes premixes and provides a cellular-connected dosifier, which is essential for good food fortification practices but is a costly piece of equipment for small-scale millers. This financial and technical support optimizes the SMEs’ resources and manufacturing processes by fortifying the flour in a homogeneous manner, allowing compliance with local regulations and avoiding the loss of inputs [54].

Sustainable funding schemes such as hybrid or blended funding models have been proposed to continue to build SMEs’ capacity in the long term [35,55]. For instance, the private sector can provide collaborative funding to SMEs in partnership with government or non-profit organizations. One example is an outcome-oriented finance scheme—a mechanism whereby the private sector provides capital to formal SMEs to buy premixes, and a third party, usually governments, returns the investment to the private sector if an SME achieves positive social outcomes [53,56,57].

In addition, SMEs can benefit from larger companies’ technical support to optimize the operation of their production and manufacturing lines and increase their revenues [58,59]. Furthermore, larger companies can guide SMEs in their business strategy by helping them to set their prices and improve their marketing and sales tactics—and thus increase their market share or expand to new outlets—and also by providing advice on nutritional claims to increase consumer demand [39,60]. Additionally, larger companies can support SMEs with guidance on safe and affordable packaging, which is essential to ensure product shelf life, food safety and an attractive packaging design—this last attribute is considered a driver for purchasing among consumers [40].

Finally, governments have a crucial role in providing an enabling environment that encourages food fortification and improves local diets. Government agencies can implement mandatory and voluntary fortification policies or adapt existing regulations by taking into consideration the nutritional context, dietary patterns, food access and availability, and challenges for food fortification. For instance, incentives such as extra SME-specific capacity training could be provided to help SMEs comply with food fortification standards [44]. In addition, governments can adjust tax policies to address the high taxation of imported micronutrient premixes [44] (i.e., offer tax breaks such as deductions, exemptions or tax returns), making it a more favorable environment for SMEs to implement food fortification practices. Additionally, there is a need to oversee and ensure adequate regulatory mechanisms and tools based on the country’s capabilities [34,47]. To address this, in collaboration with global regulatory agencies, governments could create policies, frameworks and decision trees for food fortification standards in the LMIC context. Furthermore, governments can support SMEs by implementing public food procurement (PFP) programs that represent market opportunities for them [61], and thereby help to drive the production and consumption of, and demand for, nutritious foods [62]. In the following section, we present successful case studies on how to incorporate solutions for farmers and SMEs to overcome challenges for funding, training and the regulatory environment of biofortified food.

Biofortification has been recognized as a sustainable and cost-effective approach to help tackle malnutrition globally [63,64,65,66,67]. Increasing the nutrient content of staple crops has been proven to improve nutritional status, particularly for women of reproductive age and young children in low-resource settings [67,68]. For instance, the Pearl Millet Biofortification Breeding Program has been implemented with SMEs to improve breeding capacity and distribute pearl millet crops biofortified with iron and zinc. This program resulted from a PPP between HarvestPlus, scientists from the Consultative Group for International Agricultural Research (CGIAR) at the International Crops Research Institute for the Semi-Arid Tropics (ICRISAT) and Indian Council of Agricultural Research (ICAR) [64,69].

With regard to biofortified zinc wheat flour in India, HarvestPlus has been working with SMEs to commercialize zinc wheat food products, through assisting SMEs with marketing plans and campaigns (including nutrition messaging) and catalyzing more robust supply chains that ensure affordable prices and high-quality raw materials. HarvestPlus supports SMEs with mineral analysis testing raw materials’ quality, and compliance with standards. HarvestPlus also supports SMEs to enable the environment at the local level [64,69]. For instance, by creating publicly available standards for iron and zinc [70], that can serve, in the future, to develop local biofortification standards in India.

Iron pearl millet seeds are distributed to smallholder farmers through private and public commercial channels, farmer producer groups, and community organizations. HarvestPlus develops demonstrations and informative meetings in the field or farmers’ meetings within the private channel. The demonstrations help to train, engage and provide information about the value proposition of the crop variety. HarvestPlus distributes mini-kit trial seeds to farmers to evaluate the commercial feasibility of crops. In addition, the crops are distributed by the seed companies in the regular two-tier distribution system (supply seeds to distributors, retailers and farmers). At the point of sale, trained retailers and distributors communicate to smallholder farmers the benefits of biofortified crops. In addition, the program develops demand generation strategies targeted toward farmers and final consumers, called “mobile campaigns”, to communicate the benefits of biofortified seeds. On the other hand, HarvestPlus, in partnership with the National Agricultural Research System (NARS), develops Front Line Demonstrations (FLD) of new biofortified varieties. The partners of this initiative trained more than 5500 farmers in 2021 [64,69].

Another example is a project in Rwanda that aims to scale up the production and distribution of iron-biofortified beans to reduce iron deficiency among the Rwandan population [71]. This practical, cost-effective and sustainable solution resulted from the successful public–private collaboration between The Rwanda Agriculture Board, HarvestPlus and the International Center for Tropical Agriculture. The partners developed ten iron bean varieties and distributed them among farmers. HarvestPlus established the grain aggregation center and distributed the seeds to producers. Producers distributed the iron beans within markets and improved access to consumers. In addition, apart from seed distribution, farmers were provided with agronomic training through the extension service system. This system consisted of a farmer-to-farmer approach where more than 5000 farmers and more than 1000 agricultural extension workers were trained to support smallholder farmers in cultivation techniques, marketing and the nutritional benefits of iron beans. Demand generation campaigns were also implemented to increase the uptake of iron beans among farmers and the community [71].

In summary, the iron beans project is a successful case of how innovative farmer and consumer-centric partnerships can work to achieve improved nutrition. First, the biofortification of iron beans was endorsed by the Rwandan government and standards for biofortified beans were developed by the local regulatory agency. Between 2010 and 2018, the cumulative added value of the iron beans project was estimated at USD 25 million, and approximately 5000 disability-adjusted life years (DALY) were saved due to the reduction in iron deficiency and its consequences. By 2018, over 420,000 farming households were growing iron beans, 20% of the beans produced in Rwanda were iron varieties, and 15% of Rwanda’s population (1.8 million) were consuming them. Finally, from the farmers’ perspective, they reported having 17–22% more volume production and an additional USD 57–78 of profit per hectare, as well as increasing virus resistance and heat and drought tolerance [71]. From the health and nutritional perspective, iron-biofortified beans have shown positive effects in Rwandan women, such as an increase in iron consumption and improved cognitive and physical performance (attention, memory and work efficiency) [72,73,74].

As described, SMEs can obtain assistance from various food system actors to overcome their challenges and scale up food fortification practices in LMICs. As described in the previous section, one solution that can have a positive impact and bring together multisectoral support is to build PPPs. PPPs in the nutrition field emerged as a solution to drive actions in conjunction with multiple actors, and to increase the limited public resources to address all forms of malnutrition in the population [63,64].

### 4.1. PPPs as a Possible Solution to Overcome the Burden of Malnutrition

PPPs are arrangements where public and private sectors have mutual objectives, responsibilities and economic or human resource investments to solve public health issues [75,76,77]. In the case of food fortification initiatives, PPPs can provide funding mechanisms and technical support, and advocate for the development of local government policies and regulations that can enable the environment for SMEs to be integrated as key change agents in the food supply chain. Crucial elements for effective food fortification include a business plan for self-sustainability, mandatory regulation and local standards, compliance monitoring, market research and the availability of premixes [78]. Private and public partners can support SMEs in these areas to help them optimize their food fortification practices (Figure 1) [79].

#### 4.1.1. OBAASIMA: A Case Study of a PPP for Improved Nutrition in Ghana

Micronutrient fortification has proven to be an effective intervention to tackle maternal and childhood micronutrient deficiencies [80]. However, nowadays, it is necessary to deploy solutions with a comprehensive approach that considers all forms of malnutrition. Most initiatives currently focus only on one nutritional challenge, either under- or overnutrition. Thus, especially in LMICs, where all forms of malnutrition co-exist, a shift from a simple intervention model to incorporating a DDA approach is urgently required. One example of a PPP case study is the OBAASIMA project, which aims to tackle different forms of malnutrition in Ghana.

**Figure 2:**
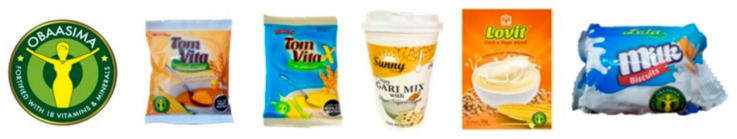
Examples of nutritious products bearing the front-of-pack OBAASIMA seal (shown left), which guarantees a product is fortified with 18 vitamins and minerals.

The OBAASIMA project incorporates a DDA approach by helping to improve the nutritional status of women of reproductive age and supporting SMEs to supply nutritious products. OBAASIMA encourages SMEs to produce nutritious foods based on a nutrient profiling (NP) framework and a front-of-pack seal that promotes micronutrient intake and limits energy-dense food consumption [81] (Figure 2). The project assists SMEs by providing technical support to help them develop new nutritious food products, facilitating the availability of vitamin and mineral premixes to fortify suitable products and testing the final products to ascertain the presence of micronutrients. Finally, social marketing campaigns were developed to increase the demand for nutritious products produced by local SMEs.

OBAASIMA was launched in 2017 as a PPP and included an initial start-up phase followed by a second scale-up phase. Local partners included the Association of Ghana Industries (AGI) and the Ghana Standards Authority (GSA). International partners included *Sight and Life*, Royal DSM, the German Development Cooperation (GIZ), the Bill & Melinda Gates Foundation, the Children’s Investment Fund Foundation and Ajinomoto. Each partner brought their expertise and capabilities to support SMEs in overcoming the barriers to engagement in fortification practices.

##### Funding

The international partners provided varying degrees of funding for different aspects of project implementation. Funding was not only direct monetary funding but also in kind, which proved essential in supporting capacity building and knowledge transfer between local and international partners.

##### Nutrient Profiling Framework

*Sight and Life* provided nutrition expertise and guided the development of an NP framework adapted to the local context. Several studies have proven that NP schemes help policymakers to develop guidance for food manufacturing [24], promote a healthy diet, prevent diseases and use front-of-pack labelling to guide consumers’ purchase decisions [82,83]. However, only a few NP schemes encourage food producers to include protein, fiber and micronutrients in their food formulations [84]. The implementation of NP schemes that consider which nutrients to limit (e.g., sugar, fat, sodium) and which nutrients to encourage (e.g., micronutrients, protein, fiber) is especially important in the LMIC context, where the intake of protein, fiber and micronutrients is low and all forms of malnutrition remain public health issues [1,33,85,86]. Thus, together with technical support and nutritional guidance, an NP framework can be the foundation to guide and encourage SMEs to produce and distribute nutritious food to help tackle the burden of malnutrition in LMICs [86,87].

##### Technical Support

Given DSM’s experience in food fortification, and as a representative of the private sector, they provide technical support to SMEs in Ghana. DSM also facilitates the availability of premixes with a micronutrient profile designed specifically for OBAASIMA products in Ghana. As a non-profit organization, *Sight and Life* provides technical guidance to SMEs to help them improve their food fortification techniques and reduce other ingredients, such as salt, sugar and fat, while fortifying their products.

##### Regulation Support

The AGI was a crucial partner in implementing the OBAASIMA seal by liaising with the Food and Drugs Authority—the authority in charge of regulating food fortification activities in Ghana—and assisting with food product certification, and the vetting and approval of food marketing materials. The AGI also recruits SMEs and encourages them to use the OBAASIMA seal on their food packaging. Finally, they also advocate for food companies to consider fortification to reduce all forms of malnutrition.

The GSA, a local government entity, was responsible for developing the OBAASIMA standards and code of practice to implement the OBAASIMA seal. The GSA allows SMEs to display a visible OBAASIMA front-of-pack seal if their products achieve the NP criteria. The OBAASIMA seal then guarantees both the targeted NP and the easy identification of fortified food products that are enriched with 18 vitamins and minerals.

##### Generating Demand

Social marketing techniques have been used to generate demand for fortified food among different communities [34]. *Sight and Life* supported the OBAASIMA project by developing formative research with women of reproductive age, and by designing and implementing a series of successful social marketing campaigns via a variety of media outlets to increase the demand for products with the OBAASIMA seal [81,88]. Results from the social marketing campaigns show that 61% of consumers recalled the advertisement about the OBAASIMA front-of-pack seal. The awareness and penetration of products with the OBAASIMA front-of-pack seal were around 30% and 5%, respectively. In addition, consumers declared that the main drivers for the consumption of OBAASIMA products were taste (79%), expiry date/shelf life (67%), health benefits (57%) and affordability (45%). Finally, 80% of consumers perceived products with the OBAASIMA seal as affordable products with good nutritional benefits; however, they struggled to find the products in different sales outlets [89]. There is therefore an opportunity for the OBAASIMA project to increase product distribution and availability at the point of sale.

From the business perspective, since being implemented, the OBAASIMA project has helped SMEs launch six products featuring the OBAASIMA seal, with sales valued above USD 1.1 million and with a considerable return on investment (ROI = 5). The project has also provided an enormous amount of learning in food fortification practices, as declared by the SMEs involved in the project. Moreover, since the implementation of the OBAASIMA project, more than 11,000 women of reproductive age have been reached with the OBAASIMA products [90], and SMEs involved in the project have been offered a new business opportunity to sell their OBAASIMA products to the World Food Programme [91]. The SMEs involved in the OBAASIMA project reported that the main challenges were the limited resources for future and sustainable marketing and advertising campaigns, and the high tax rates for the premixes [90]. This last finding reinforces our conviction about the importance of implementing sustainable funding, such as outcome-oriented schemes for SMEs.

As showcased, PPPs could create social and economic advantages, and enable the environment for SMEs to develop SBMs. Finally, from a health perspective, stakeholders involved in PPPs can guide SMEs to produce, and generate demand for, more nutritious foods and thereby help to tackle the burden of malnutrition. Consequently, this review encourages the creation of PPPs to strengthen actions towards designing and implementing strategies that consider all forms of malnutrition.

## 5. Conclusions

This review highlights the relevance of SMEs as essential actors in food fortification practices and their role in addressing all forms of malnutrition. In practice, SMEs face numerous challenges in implementing food fortification. These challenges include a lack of data and capacity building, such as financial and technical support, and the ability to comply with regulatory standards. Hence, mechanisms to overcome these challenges are crucial to building SMEs’ capacity. The case studies presented in this review advocate for a need to implement PPPs and to assist SMEs in scaling up food fortification practices to address the burden of malnutrition. Furthermore, this review suggests a holistic approach is considered while implementing food fortification strategies, by including DDA and long-term solutions to supply nutritious food in LMICs. Finally, the authors call for the implementation of a systematic reporting and monitoring system to share the results and learnings of food fortification initiatives. Such a system could help to identify the critical areas where food system actors can support SMEs and also facilitate the continued design and implementation of cost-effective food fortification strategies to tackle all forms of malnutrition.

## Data Availability

Not applicable.
